# Supplementary figures and images for: LAM Cells as Potential Drivers of Senescence in Lymphangioleiomyomatosis Microenvironment
Source: Int J Mol Sci. 2022 Jun 24;23(13):7040. doi: 10.3390/ijms23137040 (PMC9266844; doi:10.3390/ijms23137040)

Supplemental Figure S1

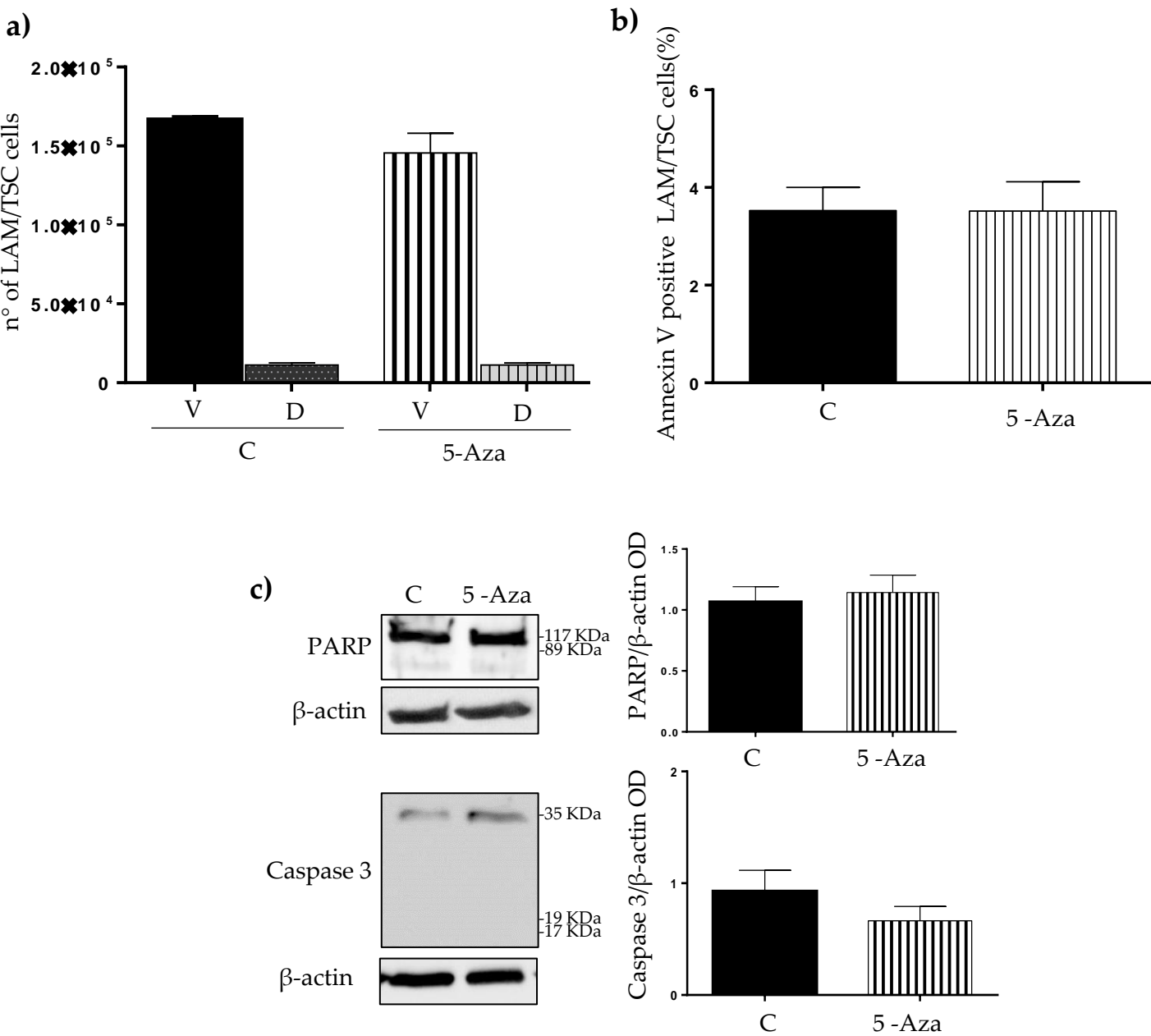

Supplemental Figure S2

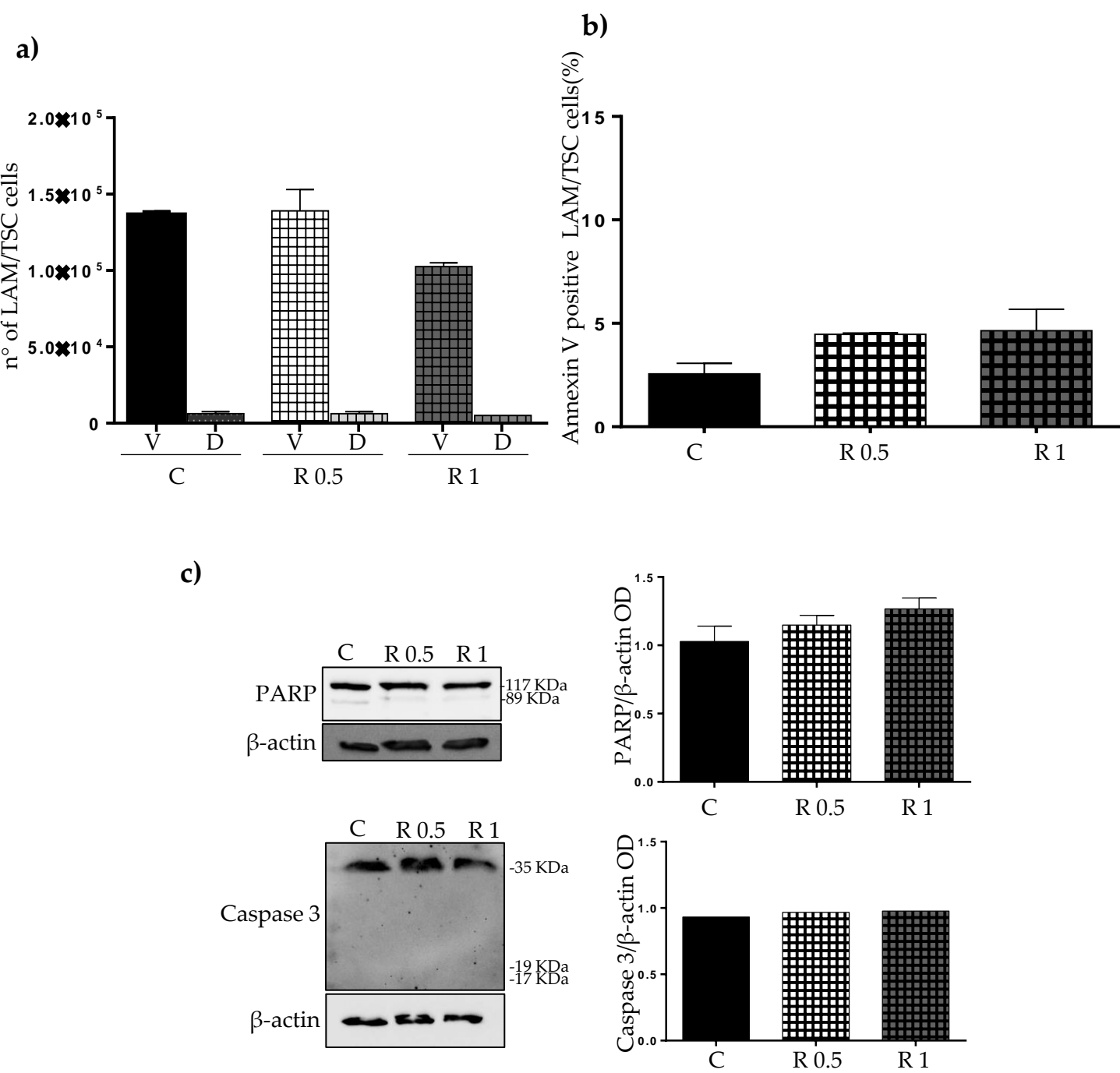

Supplement: Supplementary file 1 [file ijms-23-07040-s001.zip › ijms-1767960-supplementary.pdf]
